# Latent Syphilis Among Inpatients in an Urban Area of China

**DOI:** 10.5539/gjhs.v7n3p249

**Published:** 2014-11-30

**Authors:** Ai-Ying Liu, Wen-Jing Zang, Ling-Ling Yuan, Yong-Li Chai, ShuQi Wang

**Affiliations:** 1 Department of Dermatology, China Meitan General Hospital, Beijing, China; 2 The 260 Hospital of Shijiazhuang, China; 3 State Key Laboratory for Diagnosis and Treatment of Infectious Diseases, First Affiliated Hospital, College of Medicine, Zhejiang University, Hangzhou, China; 4 Collaborative Innovation Center for Diagnosis and Treatment of Infectious Diseases, Hangzhou, China; 5 Institute for Translational Medicine, Zhejiang University, Hangzhou, China; 6 Department of Radiology, Stanford School of Medicine, Palo Alto, CA 94304, USA

**Keywords:** latent syphilis, inpatient, sexually transmitted disease, screening

## Abstract

We aimed at investigating the epidemiological features of latent syphilis among inpatients in an urban area of China. During the period of Jan 1999 to Dec 2007, 146 inpatients were positive for treponema pallidum particle agglutination (TPPA) assay from 22,454 inpatients who were admitted to the China Meitan General Hospital. The number of latent syphilis increased steadily during this period of time. From the 146 TPPA positive inpatients, 137 inpatients were diagnosed as latent syphilis. The number of male patients with latent syphilis was slightly more than the female, but there was no statistical significance (P>0.01). The number of male patients over 60 years old was 42 (30.66%), which was higher than other age groups (p<0.05). The number of female patients at the age range of 20-29 years was 20 (14.60%), which was higher than other age groups (p<0.05). Our results demonstrated that routine syphilis screening among inpatients proves to be one of the most effective precautionary measures to identify latent syphilis and thus to prevent transmission in urban areas in China.

## 1. Introduction

In the 1950s, China implemented massive syphilis control to curb the spread of syphilis and other sexually transmitted infections (STIs) (Tucker et al., 2010). However, during the past 20 years syphilis has made a resurgence in many regions across China. It is reported in 2008 that an approximately 1 baby per hour was born with congenital syphilis in China, which had increased by 12 folds over the previous 5 years. Since syphilis infection can be asymptomatic, the screening of syphilis is of importance to disrupt transmission, particularly to reduce the infection of infants born to syphilis-infected mothers. More than 50% of pregnant women with syphilis are prone to spontaneous abortion or stillbirth. On the other hand, more than 50% of infants with congenital syphilis may suffer from irreversible sequelae and even death. In addition, studies have shown that syphilis can increase the risk of acquiring human immunodeficiency virus (HIV) infection (Bissessor et al., 2010; Chesson et al., 1999). As such, the Chinese Ministry of Health initiated a 10-year plan to control and prevent syphilis and other STIs (Tucker et al., 2011).

Several epidemiological factors have been linked to the re-emergence of syphilis in China (Tucker & Cohen, 2011; Mou et al., 2013). Rural-to-urban migration is the leading reason for rapid transmission of syphilis in China. It is estimated more than 100 million individuals migrate within China, and they tend to have a higher rate of unprotected sexual behaviors than the general population. This less-educated population is young and unmarried; the desire for commercial sex causes a hot-bed for transmission of syphilis and other STIs. Another demographic factor has been correlated with the increasing number of men who have sex with men (MSM) in China, which has been identified as a high-risk population for STIs. Although antenatal syphilis screening programs have implemented in several regions in China, none of them has reached to the national scale. The previous premarital screening program has now phased out, which increases the transmission rate of syphilis and other STIs. Similar to HIV infection, stigma exists, preventing high-risk populations from seeking sexual health services. These epidemiological factors will help identify high-risk populations, disrupt transmission route, and thus reduce the incidence.

One of the prompt issues for the prevention and control of syphilis is to detect latent infection. Currently, serological tests including nontreponemal tests for screening, and treponemal tests for confirmation. Due to the complexity of result interpretation, no single serological tests can be reliably used to diagnose syphilis and follow-up, which remains to be a challenge for rapid on-site counseling and leave subjects under-diagnosed. We here evaluated the clinical data of inpatients with latent syphilis admitted to the China Meitan General Hospital from January 1999 to December 2007, with the aim to describe the epidemiological features of inpatients with latent syphilis in the Chinese context.

## 2. Methods

### 2.1 Subjects

This is a retrospective study and we analyzed the epidemiological data from 22,454 inpatients who were admitted to the China Meitan General Hospital (Beijing) from January 1999 to December 2007. From 11,423 male patients and 11,031 female patients, 146 inpatients with positive TPPA who did not receive any anti-syphilis treatment were selected in this study. The patients’ data on age, gender and department were statistically analyzed.

### 2.2 Diagnosis of Latent Syphilis

Acquired latent syphilis was made according to the following criteria; 1) subjects were both rapid plasma reagin test (RPR) and treponema pallidum particle agglutination (TPPA) positive, 2) subjects had no blood transfusion history, 3) subjects were never diagnosed as syphilis or received any anti-syphilis treatment, 4) false positive serological reactions were excluded. Children with no syphilis symptom but positive with RPR and TPPA were diagnosed as congenital latent syphilis if their mothers had positive TPPA. In this paper, acquired latent syphilis and congenital latent syphilis were collectively defined as latent syphilis.

### 2.3 Statistical Analysis

All the data were statistically analyzed with respect to age, gender and department distribution. Data were analyzed using the SPSS12. 0 software package. The chi-square test was used to determine whether there was a significant difference in the number of latent syphilis cases between the male and female patients.

## 3. Results

### 3.1 General Status

137 inpatients who had no symptoms of syphilis were diagnosed as latent syphilis from 146 positive TPPA inpatients. Of them, 90 were males and 47 were females, and the ratio of male to female was approximately 1.10: 1. The age of diagnosis ranged from 0 to 82 years. Of 137 latent syphilis inpatients, 104 (75.9%) was married, 9 (6.6%) was divorced or widowed, and 24 (17.5%) was single. 137 inpatients with latent syphilis included 15 merchants (10.9%), 24 workers (17.5 %), 7 farmers (5.1%), 18 cadres (13.1%), 4 shop assistants (2.9%), 14 staff members (10.2 %), and 55 retirees or idlers (40.1%).

### 3.2 Incidence of Latent Syphilis From 1999-2007

The number of latent syphilis inpatients increased year by year during 1999-2007, particularly in 2007. The data showed that male with latent syphilis was slightly more than female, but there was no statistical significance (P = 0.693) (Table 1).

**Table 1:** Latent syphilis by gender from 1999-2007

	1999	2000	2001	2002	2003	2004	2005	2006	2007	Total	Percentage (‰)*
Male	1	0	1	3	2	8	14	20	23	72	52.6
Female	0	1	2	1	5	11	14	14	17	65	47.4
Total	1	1	3	4	7	19	28	34	40	137	100

* P value for the chi-square test between the male and female inpatients was 0.693.

### 3.3 Distribution of Latent Syphilis at Different Age Groups From 1999-2007

137 latent syphilis inpatients were distributed in different age groups. The number of male patients over 60 years old was 42 (30.66%), higher than other age groups. The number of female patients was 20 (14.6%) in the age range of 20-29 years and 16 (11.68%) in the age rang of 30-39, higher than other age groups (Table 2).

**Table 2:** Distribution of latent syphilis at different age groups from 1999-2007

	< 5	5~19	20~29	30~39	40~49	50~50	> 60	Total
Male	4 (2.92)	0 (0.00)	7 (5.11)	5 (3.65)	8 (5.84)	6 (4.37)	42 (30.66)	72
Female	8 (5.84)	0 (0.00)	20 (14.60)	16 (11.68)	8 (5.84)	5 (3.65)	8 (5.84)	65
Total	12 (8.76)	0 (0.00)	27 (19.71)	21 (15.33)	16 (11.68)	11 (8.03)	50 (36.50)	137

### 3.4 Distribution of Latent Syphilis at Different Departments From 1999-2007

137 latent syphilis inpatients were distributed in different departments; the cases from surgical, obstetrics and gynecology department accounted for 41.61% and 37.23%, respectively (Table 3).

**Table 3:** Distribution of latent syphilis at different departments from 1999-2007

	99	00	01	02	03	04	05	06	07	Total	Percentage (%)
Surgery	1	0	1	2	3	9	14	15	12	57	41.61
Gynecology	0	1	2	1	4	8	11	10	14	51	37.23
Inner Medicine	0	0	0	1	0	2	3	5	6	17	12.41
Cancer	0	0	0	0	0	0	0	3	6	9	6.56
Ophthalmology	0	0	0	0	0	0	0	1	2	3	2.19
Total	1	1	3	4	7	19	28	34	40	137	100

## 4. Discussion

Syphilis is caused by treponema pallidum and it is generally sexually transmitted. It has great impact on healthcare along with AIDS and other sexually transmitted diseases. Latent syphilis is diagnosed in patients with positive serologic reactions to treponema pallidum, but with no symptoms (Castro et al., 2007). Because of no clinical symptoms, patients are often under-diagnosed and result in delayed treatment, which can lead to neurological, cardiovascular, skeletal and visceral organ damage. Latent syphilis can occur when treponema pallidum initially infects human body or syphilis patients receive improper treatment (Thami et al., 2001).

In this study, a large number of latent syphilis cases were detected, when we screened for syphilis antibodies in hospitalized patients. The data showed that increasing number of latent syphilis cases in hospitalized patients from 1 case in 1999 to 40 cases in 2007 (Table 1). Although the number of latent syphilis may be under reported, there is an increase of latent syphilis associated with the increasing number of operation patients in our hospital. There were also several reports stating the increase in the number of syphilis in hospitalized patients from different urban areas in China such as Shanghai (Zhang et al., 2010), Linyi (Xiao and Wang, 2013), Luzhou (Wang et al., 2011a), and Xi’an (Sun et al., 2012). For example, the incidence of latent syphilis in inpatients in Shanxi Provincial People’s Hospital increased from 0.81% in 2008 to 1.09% in 2010 (Sun et al., 2012). In the Affiliated Hospital of Luzhou Medical College, the incidence of syphilis increased from 0.52% in 2006 to 2.74% in 2010 (Wang et al., 2011a). Although the incidence varies among different urban hospitals in China, it should be noted that there is an increasing trend of syphilis, particularly latent syphilis, occurring in China, and healthcare workers should be vigilant when caring patients for the prevention and control of syphilis.

By comparing the results of epidemiological findings of latent syphilis, we found that the positive rate of syphilis in hospitalized patients was significantly different among listed age groups (Table 2). The number of male patients in the elderly group (> 60 years old) was higher than other age groups. This unexpected finding might be contributed to the two main following reasons: 1) a sampling bias might occur since hospitalized patients cannot represent the general population. In addition, the elderly, due to reduced immunity, infection of syphilis or other diseases are more susceptible to be hospitalized than young people. 2) The high rate of latent syphilis in elderly male patients might be due to the incidence of syphilis before the founding of China, when these patients were sexually active. The distribution pattern was also observed in other studies in which people aged more than 50 years old accounted for a significant portion of latent syphilis (Sun et al., 2012; Zhang et al., 2010)

The distribution of latent syphilis among hospitalized patients varies greatly. 108 latent syphilis patients from the surgical and gynecology departments were detected, which accounted for 78.83% of total cases (Table 3). The high percentage of latent syphilis patients may be attributed to the extensive pre-operative screening of infectious diseases among surgical patients in our hospital. Another group who were positive for latent syphilis were from inner medicine department, accounting for 12.41%. Of note, the positive cases were mainly from the patients who received interventional treatment for cardiovascular diseases. These latent syphilis cases were accidentally detected through syphilis screening before transfusion. Among all the latent syphilis cases, cancer patients and ophthalmology patients accounted 6.57% and 2.19%, respectively. Since the screening of latent syphilis is not completely implemented in the hospital, the lower rates of latent syphilis might be caused by the insufficient syphilis testing in these departments. Therefore, it is necessary to increase the awareness of syphilis screening across all the departments.

In conclusion, we carried out one observational, retrospective study, investigating the incidence of latent syphilis among hospitalized patients. A consensus on syphilis testing among all the departments would increase the detection of latent syphilis. Although it might result in increased cost for patient care, a well-executed syphilis-screening program would improve clinical outcomes and help prevent transmission. Recent advances in development of microfluidics devices may offer an inexpensive solution to achieving syphilis testing at the point-of-care (Wang et al., 2011b; Wang et al., 2014).
